# A validated LC-ESI-MS/MS assay for simultaneous plasma quantification of 11 antimicrobials to support personalized dosing in critically ill children

**DOI:** 10.3389/fmolb.2026.1745458

**Published:** 2026-01-27

**Authors:** Yuan-Yuan Zhang, Jie Wang, Hong-Li Guo, Shi-Yu Wu, Hai-Feng Zhang, Peng-Fei Zheng, Hong-Jun Miao, Feng Chen

**Affiliations:** 1 Department of Pharmacy, Pharmaceutical Sciences Research Center, Children’s Hospital of Nanjing Medical University, Nanjing, China; 2 School of Basic Medicine and Clinical Pharmacy, China Pharmaceutical University, Nanjing, China; 3 Department of Orthopaedic Surgery, Children’s Hospital of Nanjing Medical University, Nanjing, China; 4 Department of Emergency and Pediatric Intensive Care Unit, Children’s Hospital of Nanjing Medical University, Nanjing, China

**Keywords:** antimicrobial, children, infection, LC-ESI-MS/MS, therapeutic drug monitoring

## Abstract

**Introduction:**

Critically ill patients with severe infections demonstrate profound alterations in pharmacokinetic behavior. Therapeutic drug monitoring (TDM)-informed antimicrobial dose optimization is thus essential in intensive care settings to ensure maximal bactericidal activity while mitigating toxicity, creating an urgent need for accessible analytical methodologies. Although various studies exist in this domain, the inherent technical constraints of current methodologies persistently prevent the full resolution of these unmet clinical requirements.

**Methods:**

A rapid and sensitive LC-ESI-MS/MS method was developed and validated for the simultaneous quantification of 11 antimicrobials in human plasma. Sample preparation was performed by a simple one-step protein precipitation using methanol containing 0.1% formic acid. The analytes were separated on a Kinetex C18 column with a “corner-folded cleaver-shaped” gradient elution program and detected using multiple reaction monitoring (MRM) in positive ionization mode.

**Results and Discussion:**

The gradient elution program achieved baseline separation of all tested analytes within 8 min, with symmetrical peak shapes and no endogenous interference. The developed method was proven to be free of matrix effects, excellent linearity (*R* ^2^ > 0.99 for all analytes), and met international bioanalytical validation criteria across clinically relevant concentration ranges. Notably, this validated method was successfully applied to children receiving mono- or combination therapy for infections. The assay meets requirements for clinical TDM implementation, providing pediatricians with reliable antibiotic concentration measurements within a clinically relevant timeframe.

## 1 Introduction

Microbial infections are a significant cause of health loss globally and are becoming the second leading cause of death worldwide (Collaborators, 2022). In reality, managing infections in critically ill patients presents a distinct and complex challenge. This complexity arises because the prevailing treatment frameworks are largely derived from infection models and clinical data that fail to adequately address the unique pharmacokinetic (PK) profiles and extreme illness severity characteristic of these individuals. Critically ill patients often exhibit profound alterations in key PK parameters—such as volume of distribution, clearance, protein binding, and tissue penetration—differing markedly from other patient populations. This mismatch frequently culminates in inadequate antimicrobial levels at both the bloodstream and the actual site of infection (Rawson et al., 2021; Tangden et al., 2017). Moreover, the rise of antibiotic resistance in various bacteria further complicates treatment and increases mortality rates, highlighting the importance of optimizing the use of antimicrobial agents to maximize therapeutic success and prolong the clinical lifespan of currently available drugs by limiting the emergence of resistance (Roberts et al., 2014). Indeed, suboptimal antimicrobial dosing frequently results in either insufficient drug exposure (leading to therapeutic failure and/or antimicrobial resistance) or excessive exposure that elevates toxicity risk (Sumi et al., 2019).

In this context, therapeutic drug monitoring (TDM), as an individualized treatment strategy, is emerging as a key tool for optimizing antimicrobial therapy. By incorporating PK monitoring, this strategy effectively enhances target concentration achievement and pharmacodynamic (PD) optimization. Notably, the IATDMCT Infectious Diseases Group formally recommended antibiotic TDM in a prior position paper, covering glycopeptides, aminoglycosides, beta-lactams (*e.g.*, penicillins, cephalosporins, monobactams, and carbapenems), and oxazolidinones (*e.g.*, linezolid) (Abdul-Aziz et al., 2020).

In particular, the clinical imperative for implementing antimicrobial TDM in pediatric populations stems from the intersection of developmental pharmacology and clinical complexity (Kearns et al., 2003). Children exhibit distinct PK/PD profiles due to ongoing maturation of drug-metabolizing enzymes, dynamic fluctuations in glomerular filtration rates, and age-dependent body composition changes. These physiological singularities are compounded by antimicrobial agents with nonlinear PK characteristics (*e.g.*, voriconazole), high interindividual variability (*e.g.*, posaconazole), and significant drug-drug interaction potential. In addition, critically ill pediatric patients with hepatic or renal impairment face further risks, as impaired drug metabolism and excretion pathways may lead to dangerous accumulation or subtherapeutic exposure. Moreover, concentration-dependent toxicities—such as imipenem-associated nephrotoxicity—necessitate rigorous dosage individualization to sustain therapeutic efficacy while minimizing adverse effects (Bruns and Dohna-Schwake, 2022; Rashed et al., 2019; Martson et al., 2020).

Suitable and accessible analytical methods are a prerequisite for implementing TDM. Indeed, literature has reported various methods to support routine TDM for antimicrobial agents. Early, Immunoassays (RIA, FIA, and ELISA) were used to measure the concentration of antimicrobial agents (Crossley et al., 1980; Hu et al., 1990; Schwenzer et al., 1983). It should be noted that the immunoassay is occasionally affected by endogenous cross-reactive substances, resulting in a false increase in measurement, and the simultaneous determination of multiple drugs is not possible (Singer et al., 2020). Afterwards, high-performance liquid chromatography (HPLC) coupled to ultraviolet detection or an evaporative light-scattering detector have been reported (Legrand et al., 2016; Verdier et al., 2011). Notably, liquid chromatography-tandem mass spectrometry (LC-MS/MS) has served as an efficient tool with high specificity and accuracy in clinical laboratories over the last 10–15 years (Shipkova and Svinarov, 2016; Li et al., 2017). Recently, various methods based on LC-MS/MS have been developed for the simultaneous determination of antimicrobial agents (Neef et al., 2024; Radovanovic et al., 2022). Still, almost all of them have a large plasma sample consumption (Lefeuvre et al., 2017; Sun et al., 2022) and complex sample clean-up methods, such as liquid-liquid extraction (Ferrari et al., 2019) and solid phase extraction (Colin et al., 2013; Ohmori et al., 2011) (Table 1), which limit the daily clinical application of the developed methods to a certain extent. The shortcomings of existing analytical methods underscore the urgent need to develop novel approaches that feature rapid sample processing, minimal sample consumption, and enhanced analytical efficiency.

**TABLE 1:** Comparison of this study with various previously published analytical methods for antimicrobial agents using the LC-MS/MS technique.

Ref	Drug	Sample volume (μL), matrix	Sample preparation	Column	Mobile phase	Range (μg/mL)	Run Time (min)
Neef et al. (2024)	10 (CEF, CIP, COLA, COLB, FLU, LZD, MEM, PRL, TAZ, and VAN)	50, plasma	PPT: ACN	ACQUITY UPLC HSS T3 C18 Column (2.1 × 100 mm, 1.8 μm)	0.1% FA in H_2_O/ACN	CEF:1–100; CIP:0.1–10; FLU:0.82–80; LZD:0.41–40; PRL:2–200; COLA:0.49–19; COLB:0.79–31; MEM, VAN:2.6–100; TAZ:1.0–40	7
Radovanovic et al. (2022)	10 (CZO, CEP, CTA, CTZ, CIP, FLU, LZD, MEM, PRL, and TAZ)	25, plasma	PPT: ACN	ACQUITY BEH C18 Column (2.1 × 50 mm; 1.7 µm)	0.1% FA in H_2_O/ACN	CZO, CEP, CTA, CTZ, and FLU:0.5–250 MEM and TAZ:0.2-100; CIP and LZD: 0.1–50 PRL:1–500	5.8
Ferrari et al. (2019)	4 (LZD, PRL, TEC, and MEM)	50, plasma	LLE: MassTox®TDM Series A basic kit	C18 column	0.1% FA in H_2_O/MeOH	PRL, MEM, and LZD:1.25–45; total TEC:1.75–63	7
Lu et al. (2022)	18 (PRL, CZO, CEF, CFP, CRO, CZO, AZT, MEM, IPM, LOF, MXF, TIG, AZM, LZD, CLI, VCZ, CAS, and VAN)	50, plasma	PPT: MeOH	ACQUITY UPLC® BEH C18 Column (2.1 × 100 mm; 1.7 µm)	0.1% FA in H_2_O/ACN	PRL:2.18–217.8; CZO:2.14–214; CEF:2.16–216; CFP:2.4–240; CRO:2.2–220; CEP:2.22–222; AZT:2.15–214.5; MEM:2.02–202; IPM:1.99–199; LOF:0.55–55.13; MXF:0.28–28.44; TIG:0.06–5.76; AZM:0.22–21.7; LZD:0.39–38.5; CLI:0.22–21.56; VCZ:0.1–10.2; CAS:0.12–12; VAN:0.41–40.55	6
Colin et al. (2013)	10 (AMO, AMP, FEN, PRL, CEF, CDX, FLU, MEM, CFP, CYZ, TAZ, LZD, and CFZ)	50, plasma	SPE: Oasiss MCX μ-elution 96-well plates	ACQUITY HSS T3 C18 column (50 × 2.1 mm, 1.7 µm)	1 mM AA/NH_4_AC-buffer with 5% ACN in H_2_O/ACN	AMO:0.43–51.05; AMP:0.58–70.05; FEN:0.06–7.28; PRL:0.32–38.06; CEF:0.48–57.69; CDX:0.05–6.04; FLU:0.08–9.11; MEM:0.17–20.06; CFP:0.18–21.53; CYZ:0.76–90.81; TAZ:0.24–28.24; LZD:0.05–5.94; CFZ:0.61–73.01	4
Barco et al. (2020)	15 (AMK, AMO, CTZ, CIP, COLIA, COLIB, DAP, GEN, LZD, MEM, PRL, TEC, TIG, TOB, VAN, and TAZ)	50, plasma	PPT: 10% trichloroacetic acid/MeOH	ACCUCORE™ Polar Premium column (50 × 2.1 mm; 2.6 µm)	0.1% FA in H_2_O/ACN	AMK, AMO, GEN, LZD, TOB, and TAZ:0.4–40 CTZ, DAP, and PRL:2.0–200; MEM, TIG, VAN, and TEC:1.0–40; CIP:0.1–10; COLA:0.3–26; COLB:0.5–54	5
Lefeuvre et al. (2017)	15 (AMO, CEP, CTA, CTZ, CRO, CIP, CLI, ETP, IPM, MEM, LOF/OF, OXA, PRL, TIC, and TAZ)	100, plasma	PPT: 0.1% FA in MeOH	ACCUCORE C18 column (100 × 2.1 mm; 2.6 µm)	2 mM AF with 0.1% FA in H_2_O/0.1% FA in ACN	0.5–32	9
Ohmori et al. (2011)	8(CFZ, CEP, CMZ, MEM, AMP, CTA, DOR, and PRL)	50, serum	SPE: Waters Oasis® HLB cartridges	Unison UK-C18 column (50 × 2 mm; 3 µm)	10 mM AF with 0.1% FA in H_2_O/0.1% FA in MeOH	CFZ, CEP, CMZ, MEM, AMP, CTA, PRL:0.1–50 DOR:0.5–50	8
Sun et al. (2022)	14 (MEM, NOR, AMP, LZD, CTZ, CRO, CFP, VAN, CEP, BIA, MOX, ETI, DAP, and AMK)	100, plasma/CSF	PPT: ACN-MeOH (3: 1, v/v)	ACQUITY UPLC BEH C18 column (2.1 × 50 mm; 1.7 μm)	0.1% FA in H_2_O/MeOH	MEM, NOR, AMP, and DAP:0.5–100; LZD:0.25–50; CTZ:0.4–80; CRO:0.05–10; CFP and VAN:0.5–50; CEP:0.2–20; BIA:0.25–25; MOX:0.6–60; ETI:0.65–40; AMK:1.3–80	3
This study	11 (MOX, VCZ, CAS, CMZ, LZD, MEM, IPM, TIG, CFZ, PRL, and POS)	10, plasma	PPT: 0.1% FA in MeOH	Kinetex C18 column (2.1 × 75 mm; 2.6 μm)	0.1% FA in H_2_O/ACN	IPM, MEM, MOX, PRL, CMZ, and CFZ:1–100 CAS, POS, and TIG:0.5-50; LZD:0.25-25; VCZ:0.1–10	6.5

Abbreviations: AA, acetic acid; AF, ammonium formate; AMK, amikacin; AMO, amoxicillin; AMP, ampicillin; AZT, aztreonam; AZM, azithromycin; BIA, biapenem; CAS, caspofungin; CDX, cefadroxil; CEF, cefuroxime; CEP; cefepime; CFP, cefoperazone; CFZ, cefazolin; CIP, ciprofloxacin; CLI, clindamycin; CMZ, cefmetazole; COLA, Colistin A; COLB, Colistin B; CRO, ceftriaxone; CSF: cerebrospinal fluid; CTA, cefotaxime; CTZ, ceftazidime; CZO, cefazolin; DAP, daptomycin; DOR, doripenem; ETP, ertapenem; ETI, etimicin; FEN, phenoxymethylpenicillin; FLU, flucloxacillin; GEN, gentamicin; IPM, imipenem; LOF, levofloxacin; LZD, linezolid; MEM, meropenem; MOX, latamoxef; MXF, moxifloxacin; NOR, norvancomycin; OXA, oxacillin; PRL, piperacillin; TAZ, tazobactam; TEC, teicoplanin; TIC, ticarcillin; TIG, tigecycline; TOB, tobramycin; VAN, vancomycin; VCZ, voriconazole.

Therefore, this study aimed to develop and validate a LC-electrospray (ESI)-MS/MS method for simultaneous analysis of 11 antimicrobial agents (ATBs; *i.e.*, caspofungin, imipenem, linezolid, meropenem, latamoxef, piperacillin, voriconazole, posaconazole, cefmetazole, tigecycline, and cefazolin) in human plasma and to investigate its applicability for routine TDM in clinical practice in children with microbial infections. Of note, this study successfully determined the 11 ATBs simultaneously with minimum run time (6.5 min) and small-volume plasma consumption (10 μL). Impressively, our method minimized medical costs by optimizing extraction processing and enabled the analysis with a simple one-step protein precipitation (0.1% FA in MeOH) for sample pretreatment. The fast and efficient method provides valuable concentration data for patients and pediatricians.

## 2 Experimental methods

### 2.1 Materials

Latamoxef (MOX) and voriconazole (VCZ) were purchased from the National Institutes for Food and Drug Control (Beijing, China). Caspofungin (CAS), cefmetazole (CMZ), linezolid (LZD), meropenem (MEM), imipenem (IPM), tigecycline (TIG), linezolid-d3 (LZD-d3), piperacillin-d5 (PRL-d5), voriconazole-d3 (VCZ-d3), meropenem-d6 (MEM-d6), and caspofungin acetate-d4 (CAS-d4) were obtained from Toronto Research Chemicals Inc. (Toronto, Canada). Cefazolin (CFZ) was purchased from Anpel-Trace Standard Technical Services (Shanghai) Co., Ltd. Piperacillin (PRL) was supplied by TMstandard® (Beijing, China). Posaconazole (POS) was purchased from LGC Standards (Molsheim, France). HPLC-grade methanol (MeOH) and acetonitrile (ACN) were purchased from Merck KGaA (Darmstadt, Germany). Isopropanol (IPA) of HPLC grade was supplied by Fisher Scientific (Fairlawn, USA). LC-MS grade formic acid (FA) was obtained from TCI (Shanghai, China) Development Co., Ltd. ACS-grade ammonium acetate (NH_4_AC) was obtained from Sigma-Aldrich Co. LLC (Wilmington, United States). Ultrapure water was prepared using a Milli-Q water purification system (Millipore, Bedford, USA).

Chromatographic columns including Kinetex C8 100 Å (2.1 mm × 50 mm, 5 μm), Kinetex C18 100 Å (2.1 mm × 50 mm, 1.7 μm), Kinetex C18 100 Å (2.1 mm × 50 mm, 2.6 μm), Kinetex C18 100 Å (2.1 mm × 50 mm, 5 μm), Kinetex C18 100 Å (2.1 mm × 100 mm, 2.6 μm), Luna C18 (2) 100 Å (2 mm × 50 mm, 5 μm), Gemini C18 110 Å (2 mm × 50 mm, 3 μm), and Synergi™ Fusion-RP 80 Å (2 mm × 50 mm, 4 μm) were purchased from the Phenomenex Inc. (Torrance, CA, USA). The Waters Symmetry C18 column (4.6 mm × 75 mm, 3.5 μm) was obtained from the Waters Corporation (Milford, MA, USA). A column designed for hydrophilic interaction chromatography (HILIC) (4.6 mm × 50 mm, 3 μm) was purchased from the OSAKA SODA CO., LTD. (Osaka, Japan).

### 2.2 Instruments and conditions

Chromatographic separation and analyte quantification were performed using a Jasper™ LC system (AB Sciex Pte. Ltd., Singapore) coupled to a Triple Quad™ 4500MD mass spectrometer (AB Sciex Pte. Ltd., Singapore). The LC system comprised a binary pump (Sciex Dx™), an online degasser (Sciex Dx™), an autosampler (Sciex Dx™), and a thermostated column compartment (Sciex Dx™).

Chromatographic separation was performed on a Kinetex C18 (2.1 mm × 75 mm, 2.6 μm; Phenomenex) protected by a security Guard-C18 column (4 × 2.0 mm; Phenomenex). A gradient elution program was employed using: (A) 0.1% formic acid in H_2_O and (B) 0.1% FA in ACN at a mixed flow rate of 0.25 mL/min and 0.5 mL/min, respectively. The gradient profile was:

0–0.5 min: 8% B,

0.5–1 min: 8→50% B,

1–3.5 min: 50→85% B,

3.5–4.2 min: 85→90% B,

4.2–4.7 min: 90% B,

4.7–4.8 min: 90→8% B,

4.8–6.5 min: 8% B.

The column was maintained at 45 °C and autosampler at 4 °C, with an injection volume was 5 μL.

Quantification was conducted on a Triple Quad™ 4500MD mass spectrometer (AB Sciex Pte. Ltd., Singapore) equipped with an ESI source operated in positive/negative ion switching mode. Optimized multiple reaction monitoring (MRM) parameters for each analyte are provided in Table 2. Key source parameters including curtain gas (CUR), collision-activated dissociation (CAD), temperature (TEM), nebulizer gas (GS1), and heater gas (GS2) were set as 20 psi, 6 units, 450 °C, 50 psi, and 50 psi, respectively. The ion spray voltage was maintained at 5500 V in positive ion mode. Data acquisition and peak processing were performed using Analyst MD software (version 1.6.3; AB Sciex Pte. Ltd., Singapore).

**TABLE 2:** Multiple reaction monitoring parameters, linear, IS, and chromatographic retention times for the 11 ATBs tested in this study.

Analyze	Ionization mode	Precursor (m/z)	Product (m/z)	DP (v)	EP (v)	CE (v)	CXP (v)	Linear range (μg/mL)	Linear equation (w = 1/x^2^)	R^2^ value	RT (min)	IS
CAS	[M+2H]^2+^	547.3	137.2	110	7	35	10	0.5–50	y = 0.9914x + 0.01129	0.9972	1.86	CAS-d4
IPM	[M + H] ^+^	299.9	141.9	68	10	40	12	1–100	y = 0.9308x + 0.6299	0.9931	1.42	VCZ-d3
LZD	[M + H] ^+^	338.2	296.2	110	11	16	12	0.25–25	y = 0.9856x + 0.0285	0.9993	1.87	LZD-d3
MEM	[M + H] ^+^	384.3	141.0	84	10	20	10	1–100	y = 0.9486x + 0.4619	0.9906	1.71	MEM-d6
MOX	[M + H] ^+^	520.9	377.0	90	7	20	15	1–100	y = 1.045x - 0.4060	0.9943	1.79	VCZ-d3
PRL	[M + H] ^+^	518.3	143.0	106	10	49	10	1–100	y = 0.9830x + 0.1375	0.9931	1.93	PRL-d5
VCZ	[M + H] ^+^	350.0	280.8	90	13	35	11	0.1–10	y = 0.9682x + 0.030	0.9980	2.12	VCZ-d3
POS	[M + H] ^+^	701.3	683.4	1	2	43	22	0.5–50	y = 1.069x - 0.3733	0.9979	2.34	VCZ-d3
CMZ	[M + H] ^+^	472.1	356.0	17	6	12	11	1–100	y = 0.9401x + 0.5295	0.9949	1.84	PRL-d5
TIG	[M+2H]^2+^	293.7	257.1	75	11	18	20	0.5–50	y = 0.9283x + 0.3540	0.9958	1.68	VCZ-d3
CFZ	[M + H] ^+^	455.1	323.0	100	10	16	10	1–100	y = 0.9261x + 0.6735	0.9955	1.80	PRL-d5
IS												
CAS-d4	[M+2H]^2+^	549.4	141.0	105	9	45	10				1.86	
LZD-d3	[M + H] ^+^	341.2	297.1	120	10	28	13				1.87	
MEM-d6	[M + H] ^+^	390.0	147.2	80	8	24	10				1.71	
PRL-d5	[M + H] ^+^	523.0	148.3	107	8	30	8				1.93	
VCZ-d3	[M + H] ^+^	353.0	284.0	90	10	23	13				2.11	

Abbreviations: CE, collision energy; CXP, collision cell exit potential; DP, declustering potential; EP, entrance potential; IS, internal standard; RT, retention time.

### 2.3 Preparation of stock solutions, calibration standards, and quality control (QC) samples

Stock solutions of CAS, CFZ, CMZ, MOX, PRL, VCZ, and MEM were prepared in MeOH at 10.00 mg/mL, except for POS and LZD prepared in 5.00 mg/mL. Stock solutions of IPM were dissolved in H_2_O at 5.00 mg/mL, and 8.00 mg/mL for TIG in MeOH, respectively. The working solutions of antibiotics were further prepared by diluting the stock solution in 50% MeOH to obtain the target concentrations. All the stock solutions and working solutions were stored at −80 °C. Internal standard (IS) stock solutions of PRL-d5, CAS-d4, MEM-d6, LZD-d3, and VCZ-d3 were prepared in MeOH to give a concentration of 1.00 mg/mL. A mixed IS working solution was prepared in MeOH containing CAS-d4 and MEM-d6 at 1,000 ng/mL, LZD-d3 and VCZ-d3 at 50.0 ng/mL, and PRL-d5 at 100 ng/mL, respectively.

Calibration standards were prepared in duplicate by spiking 10.0 μL of the standard working solutions into 10.0 μL blank plasma. Final concentrations covered the following ranges:

- IPM, MEM, MOX, PRL, CMZ, CFZ: 1.00, 2.00, 4.00, 10.0, 20.0, 60.0, 100.0 μg/mL
- CAS, POS, TIG: 0.500, 1.00, 2.00, 5.00, 10.0, 30.0, 50.0 μg/mL
- LZD: 0.25, 0.50, 1.00, 2.50, 5.00, 15.0, 25.0 μg/mL
- VCZ: 0.100, 0.200, 0.400, 1.00, 2.00, 6.00, 10.0 μg/mL

Calibration curves were generated by plotting the analyte-to-internal standard peak area ratio versus the nominal standard concentrations.

QC samples were prepared in the same way as calibration standards at 4 concentration levels:

•1.00 μg/mL (the lower limit of quantification QC, LLOQ QC), 3.00 μg/mL (low QC, LQC), 16.0 μg/mL (medium QC, MQC), 80.0 μg/mL (high QC, HQC) for IPM, MEM, MOX, PRL, CMZ, and CFZ;
- 0.500, 1.50, 8.00, and 40.0 μg/mL for CAS, POS, and TIG;
- 0.250, 0.750, 4.00, and 20.0 μg/mL for LZD;
- 0.100, 0.300, 1.60, and 8.00 μg/mL for VCZ.

### 2.4 Sample preparation

Calibration standards, QC samples, and plasma samples were processed via protein precipitation: for calibrators/QCs, a mix of 10.0 μL working solutions and 10.0 μL blank plasma was added to 80.0 μL of a MeOH/FA mixture (99.9/0.1; v/v), while plasma samples used a mix of 10.0 μL plasma sample and 10.0 μL MeOH added to 80.0 μL identical precipitant. Then, all mixtures were vortexed for 5 min and centrifuged for 5 min at 4,000 rpm at 4 °C. An aliquot of 20.0 μL of the supernatant was transferred to another clean 1.5 mL Eppendorf tube and diluted with 180 μL of H_2_O:ACN (95:5; v/v). Next, the mixtures were vortexed well for 3 min and a 5 μL aliquot of the diluted supernatant was injected for LC-ESI-MS/MS analysis. Drug-free human plasma was obtained from the Department of Blood Transfusion, Children’s Hospital of Nanjing Medical University.

### 2.5 Method validation

Method validation was conducted in accordance with the US Food and Drug Administration Bioanalytical Method Validation Guidance for Industry (2018) (U.S. Food and Drug Administration, 2018).

#### 2.5.1 Selectivity and carryover

The double blank plasmas from six sources were tested to investigate the selectivity of the method. Furthermore, the interference between ATBs and IS was assessed using the upper limit of quantification (ULOQ) without IS and control blank samples (only IS working solution was spiked into blank plasma). The interference between each analyte was also evaluated by analyzing blank plasma samples spiked with single ATB at ULOQ.

In addition, carryover was assessed by injecting blank plasma samples immediately after the ULOQ calibration of standard samples.

#### 2.5.2 Linearity and LLOQ

Linearity was constructed by the peak area ratio of ATBs to IS versus the concentrations of the calibration standards, and the calibration curves in the form of y = a + bx were processed by weighted (1/*x* ^2^) least-squares linear regression. The LLOQs are considered the lowest concentrations on the calibration curves and were based on a signal-to-noise ratio (S/N) of 5, at least. The intra- and inter-day accuracy and precision of LLOQs should be less than 20% each.

#### 2.5.3 Accuracy and precision

Method accuracy and precision were validated through six replicates at four QC levels (LLOQ, LQC, MQC, and HQC). Intra-day precision was evaluated in a single analytical batch, while inter-day precision was evaluated in three consecutive batches. Dilution integrity was additionally demonstrated using diluted QC (DQC). The accuracy of the method was expressed as relative error (RE%) and the precision was calculated as the relative standard deviation (RSD%).

#### 2.5.4 Recovery and matrix effect

Recovery of the analyte at the three QC levels by comparing the ratio of peak areas from the regularly prepared samples with that from the spike-after-extraction samples with six replicates per level. To prepare the spike-after-extraction samples, a series of intermediate concentration (named “INT” concentration) points was formulated. Briefly, the blank sample was processed according to the sample preparation procedure described above. The supernatant was mixed with an “INT” concentration of ATBs at concentrations corresponding to the final concentration of the pretreated plasma samples. The extraction recovery of the internal standard was determined similarly. The matrix effect was evaluated by calculating the ratio of peak areas of analyte spike-after-extraction (six different sources of blank sample) to an equivalent concentration of the same analyte standard in H_2_O:ACN (95:5; v/v). IS-normalized matrix factors were used to evaluate the matrix effect.

#### 2.5.5 Stability

The stability in plasma was performed using triplicate QC samples at two concentration levels (LQC and HQC) of each analyte. Samples were exposed to different storage conditions: (1) bench-top stability (at room temperature), (2) freeze and thaw stability (five freeze-thaw (−20 °C) cycles), (3) long-term stability (at −20 °C), and (4) autosampler stability (in autosampler at 4 °C).

### 2.6 Application to clinical samples for routine TDM

To demonstrate the applicability of the method, the validated method was applied to pediatric patients at the Children’s Hospital of Nanjing Medical University, who were treated with one or more antibiotics included in this assay. Blood samples were collected in EDTA anticoagulant tubes during steady-state concentrations (24–48 h post-treatment initiation). The study complied with the Declaration of Helsinki and received ethical approval from the Children’s Hospital of Nanjing Medical University ethics committee.

## 3 Results

### 3.1 Method development

#### 3.1.1 Optimization of mass spectrometry

**FIGURE 1:**
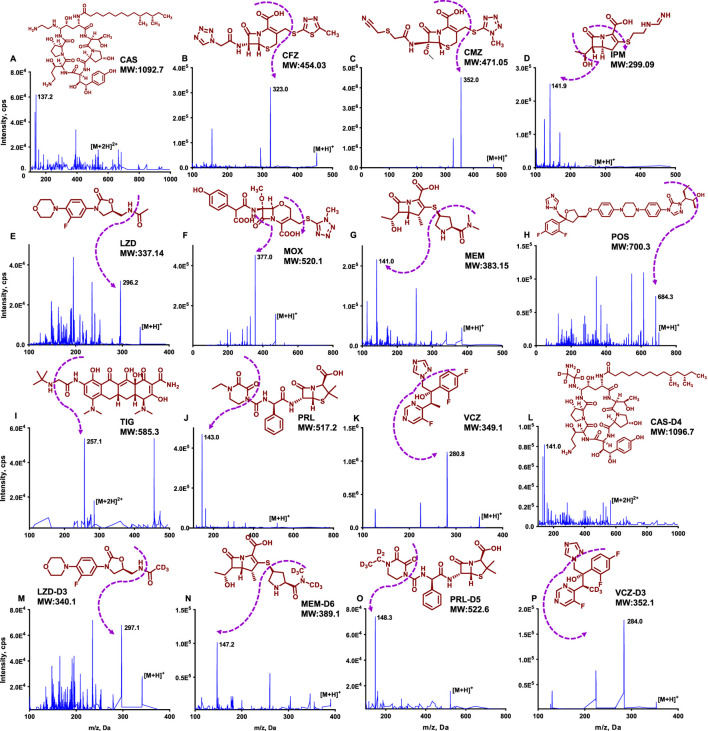
The MS2 chromatograms for 11 ATBs and ISs.

Using a syringe pump, ESI and atmospheric pressure chemical ionization (APCI) were compared to obtain a more suitable ionization mode. All ATBs generated the protonated molecular ion in positive-ion mode in full scan with a m/z range from 100 to 2000. TIG and CAS were monitored with [M + 2H]^2+^ species, and other ATBs were monitored with [M + H]^+^ species. The MRM transitions with the best S/N for ATBs and internal standard were presented in Table 2, and the MS2 spectra are shown in Figure 1. The sensitivity of the signal was largely dependent on the mass spectrometric parameters for the ESI process. The CUR (from 25 to 40 psi), CAD (from 4 to 10 units), IS (from 4,500 to 5,500 V), GS1 (from 40 to 55 psi), GS2 (from 40 to 55 psi), and TEM (from 450 °C to 550 °C) were optimized to obtain the highest intensity of protonated molecules. Similarly, parameters like dwell time, declustering potential (DP), entrance potential (EP), collision energy (CE), and collision cell exit potential (CXP) were also optimized. The MS operating parameters are summarized in Table 2.

#### 3.1.2 Liquid chromatographic conditions optimization

In this study, the chromatographic columns of different particle sizes (from 1.7 to 5 μm), lengths (from 50 to 100 mm), and packing materials (core-shell particles and porous particles) were compared to obtain better separation, peak shapes, and resolution. Finally, the Kinetex C18 (2.1 × 75 mm, 2.6 μm) column provided better chromatographic separation and peak shapes. The effects of column temperature and flow rate are also taken into account. The high abundance and peak shape were observed at a column temperature of 45 °C and a mixed flow rate of 0.25/0.5 mL/min. A methanol formic acid mixture (99.9/0.1; v/v) as precipitant could achieve a good resolution and lower background noise.

**FIGURE 2:**
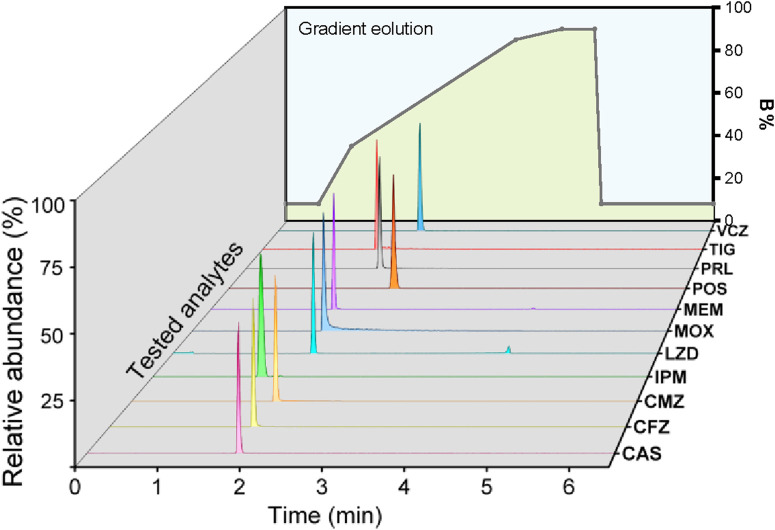
The “corner-folded cleaver-shaped” gradient elution program for 11 ATBs. Under this gradient condition, the chromatographic peak pattern of each antibiotic.

Considering the wide polarity distribution of antimicrobial agents, a gradient elution instead of isocratic elution was utilized to obtain narrow and symmetrical peak shapes. A “corner-folded cleaver-shaped” gradient elution was established, with an initial B-phase set at 8% so that the carry-over of TIG and MOX was reduced (Figure 2). However, significant carry-over was still observed. As a result, a complex needle wash solution containing ACN: MeOH: IPA: H_2_O (4:4:1:1, v/v/v/v) was used to decrease the carry-over.

Different concentrations of FA and NH_4_AC were added to the mobile phase. Results showed that the carry-over of TIG and MOX significantly decreased when the FA concentrations increased. In addition, different concentrations of NH_4_AC showed minimal impact on the chromatographic performance of the analytes. Thus, the addition of 0.1% FA to H_2_O/ACN was ultimately selected as the mobile phase.

### 3.2 Method validation

#### 3.2.1 Selectivity and carryover

**FIGURE 3:**
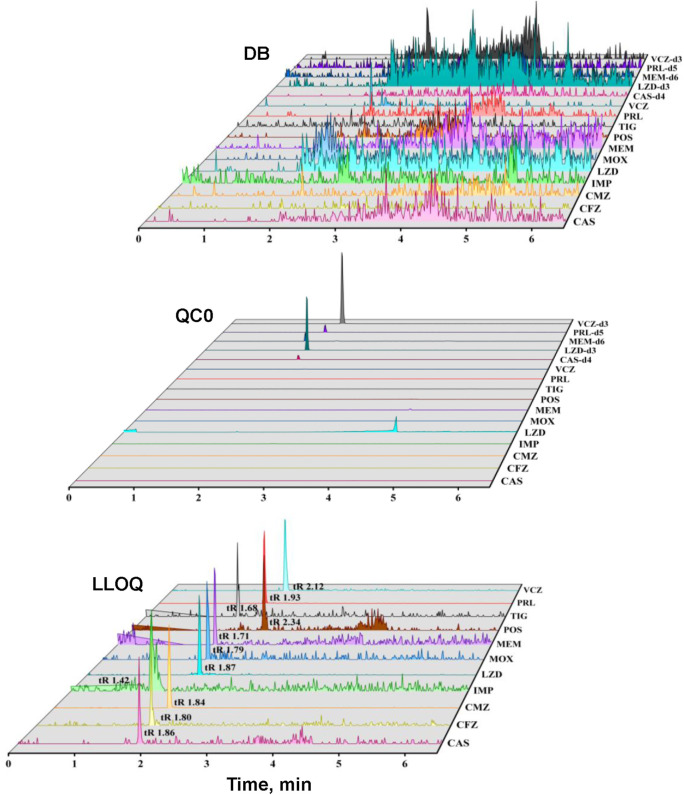
Representative chromatograms of double blank sample, IS of each ATB, LLOQ of 11 ATBs (Caspofungin, CAS; Cefazolin, CFZ; Cefmetazole, CMZ; Imipenem, IPM; Linezolid, LZD; Latamoxef, MOX; Meropenem, MEM; Posaconazole, POS; Tigecycline, TIG; Piperacillin, PRL; Voriconazole, VCZ; Caspofungin Acetate-d4, CAS-d4; Linezolid-d3, LZD-d3; Meropenem-d6, MEM-d6; Piperacillin-d5, PER-d5; Voriconazole-d3, VCZ-d3).

The representative MRM chromatograms of the double blank sample (A) and the QC sample of 11 ATBs (B) are shown in Figure 3. No significant interference was found at the retention times of each analyte. Moreover, no interference was observed between each analyte.

Additionally, the carry-over was defined as the percentage of responses from the blank sample to the respective LLOQ sample. No significant carry-over was observed for the tested ATBs, except for MOX in the system (Table 3).

**TABLE 3:** The selectivity and carry-over of 11 ATBS and ISs.

Analytes	Selectivity	IS *VS*. ATBs (%)	ATBs *VS*.IS (%)	Carry-over (%)
CAS	6.0	2.4	4.5	11.7
IPM	4.2	0.8	4.5	11.3
LZD	8.4	5.0	16.6	11.1
MEM	8.7	2.0	1.1	2.4
MOX	3.5	0.5	4.5	18.9
PRL	1.0	0.1	1.3	5.6
VCZ	12.6	0.1	4.7	16.9
POS	10.5	2.3	4.7	16.6
CMZ	1.9	0.2	4.7	6.4
TIG	13.8	7.0	3.4	5.5
CFZ	3.4	0.8	4.5	10.8
IS				
CAS-d4	1.1			1.1
VCZ-d3	1.0			1.1
LZD-d3	8.6			9.7
MEM-d6	1.2			0.1

#### 3.2.2 Linearity and LLOQ

The typical regression equations of the calibration curves are listed in Table 2. The linearity of the calibration was satisfactory over the concentration range for all the compounds, with correlation coefficients higher than 0.99. The representative chromatograms of LLOQ (S/N > 5) are shown in Figure 3, and the accuracy in plasma ranged from 88.5% to 108.0%, and the RSD of precision was less than 13.3%. The results indicated that this method was accurate and reliable.

**FIGURE 4:**
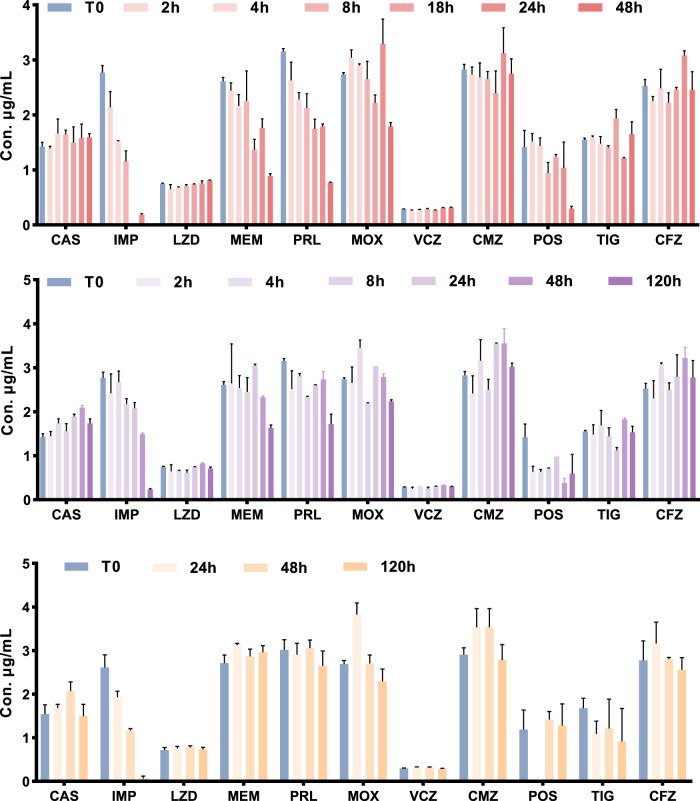
The stability of 11 antimicrobial agents under different storage conditions. A at room temperature. B in a 4 °C refrigerator. C in the autosampler at 4 °C after extraction.

#### 3.2.3 Accuracy and precision

The intra- and inter-day accuracy and precision data are listed in Table 4. The intra-day precision ranged from 2.9% to 10.2%, and the mean accuracy values ranged from 90.7% to 111.8%. The inter-day precision and mean accuracy ranged from 4.0% to 10.3% and from 92.5% to 108.3%. The results demonstrated that the method was accurate and precise (RSD <15%, RE within ±15%).

**TABLE 4:** Intra- and inter-day accuracy and precision of the LC-MS/MS method for the 15 ATBs used in this study (n = 6).

Analytes	LLQC (μg/mL)		Precision	Accuracy	LQC (μg/mL)		Precision	Accuracy	MQC (μg/mL)		Precision	Accuracy	HQC (μg/mL)		Precision	Accuracy
			(RSD %)	(RE %)			(RSD %)	(RE %)			(RSD %)	(RE %)			(RSD %)	(RE %)
Intraday																
CAS	0.50	0.47 ± 0.03	7.1	−6.6	1.50	1.46 ± 0.05	3.4	−2.7	8.00	7.62 ± 0.34	4.5	−4.8	40.00	37.77 ± 2.06	5.6	−5.5
IPM	1.00	0.99 ± 0.10	9.9	−1.2	3.00	3.23 ± 0.33	10.2	7.7	16.00	15.90 ± 1.00	6.3	−0.6	80.00	75.98 ± 6.01	7.9	−5.0
LZD	0.25	0.22 ± 0.01	6.3	−11.2	0.75	0.76 ± 0.05	6.9	0.9	4.00	4.11 ± 0.37	9.0	2.8	16.00	18.87 ± 0.90	4.8	−5.5
MEM	1.00	1.04 ± 0.09	8.8	4.4	3.00	2.89 ± 0.26	9.0	−3.7	16.00	15.20 ± 1.01	6.6	−5.0	80.00	78.22 ± 4.66	6.0	−2.3
MOX	1.00	1.05 ± 0.09	9.1	4.9	3.00	2.99 ± 0.13	4.3	−0.3	16.00	16.70 ± 0.75	4.8	4.4	80.00	89.40 ± 3.24	3.6	11.8
PRL	1.00	0.93 ± 0.09	9.4	−7.2	3.00	3.06 ± 0.28	9.7	3.3	16.00	16.37 ± 1.43	8.5	2.5	80.00	83.08 ± 6.13	7.3	3.9
VCZ	0.10	0.09 ± 0.01	12.1	−0.8	0.30	0.31 ± 0.02	5.5	3.0	1.60	1.56 ± 0.12	7.7	−2.5	8.00	7.81 ± 0.36	4.6	−2.4
POS	0.50	0.47 ± 0.05	10.5	−6.6	1.50	1.51 ± 0.12	7.9	0.7	8.00	7.52 ± 0.52	6.9	−6.0	40.00	39.52 ± 3.61	9.1	−1.3
CMZ	1.00	1.05 ± 0.10	9.4	5.0	3.00	3.28 ± 0.15	4.6	9.3	16.00	16.97 ± 0.52	2.9	6.3	80.00	84.02 ± 4.68	5.6	5.0
TIG	0.50	0.54 ± 0.05	10.0	8.0	1.50	1.45 ± 0.08	5.5	−3.3	8.00	7.75 ± 0.62	8.0	−3.1	40.00	35.85 ± 1.72	4.8	−10.3
CFZ	1.00	0.89 ± 0.05	5.8	−11.5	3.00	3.06 ± 0.19	6.2	2.0	16.00	14.95 ± 0.89	6.0	−6.3	80.00	79.32 ± 4.94	6.2	−0.9
Interday																
CAS	0.50	0.45 ± 0.06	12.0	−3.2	1.50	1.48 ± 0.07	4.7	−1.3	8.00	8.10 ± 0.51	6.3	1.3	40.00	38.95 ± 2.61	6.7	−2.5
IPM	1.00	0.99 ± 0.10	10.4	−0.7	3.00	3.10 ± 0.32	10.3	3.3	16.00	15.25 ± 1.11	7.2	−4.4	80.00	74.89 ± 6.86	9.2	−6.4
LZD	0.25	0.23 ± 0.02	9.1	−7.2	0.75	0.78 ± 0.05	6.3	4.5	4.00	4.26 ± 0.26	6.1	6.5	16.00	19.94 ± 1.21	6.0	−0.5
MEM	1.00	1.04 ± 0.10	9.2	4.4	3.00	3.00 ± 0.22	7.3	0.0	16.00	15.53 ± 1.22	7.7	−3.1	80.00	77.46 ± 4.96	6.5	−3.1
MOX	1.00	1.07 ± 0.09	8.9	6.6	3.00	2.96 ± 0.20	6.8	−1.3	16.00	16.34 ± 0.74	4.3	1.9	80.00	85.38 ± 4.68	5.5	6.8
PRL	1.00	1.00 ± 0.09	8.6	0.6	3.00	3.10 ± 0.23	7.4	3.3	16.00	16.25 ± 1.01	6.2	1.3	80.00	81.84 ± 6.22	7.6	2.3
VCZ	0.10	0.10 ± 0.01	7.9	−3.0	0.30	0.31 ± 0.02	5.5	2.3	1.60	1.60 ± 0.09	5.6	0.0	8.00	7.95 ± 0.32	4.0	−0.6
POS	0.50	0.51 ± 0.05	10.0	2.0	1.50	1.45 ± 0.09	6.2	−3.3	8.00	7.77 ± 0.45	5.8	−2.9	40.00	40.85 ± 3.62	8.8	2.3
CMZ	1.00	1.00 ± 0.10	9.9	0.4	3.00	3.25 ± 0.16	4.9	8.3	16.00	17.12 ± 0.82	4.7	6.9	80.00	81.86 ± 5.34	6.5	2.4
TIG	0.50	0.51 ± 0.06	12.0	1.8	1.50	1.52 ± 0.11	7.2	1.3	8.00	7.96 ± 0.58	7.3	−0.5	40.00	37.00 ± 2.78	7.5	−7.5
CFZ	1.00	0.90 ± 0.12	13.3	−10.0	3.00	3.01 ± 0.27	9.0	0.3	16.00	15.91 ± 0.95	6.3	−0.6	80.00	78.08 ± 5.43	6.9	−2.4

Abbreviations: Con., concentration; n, number of replicates; RE, relative error; RSD, relative standard deviation.

#### 3.2.4 Recovery and matrix effect

Table 5 presents the recovery and matrix effects of analytes. The RSD of matrix effects derived from QC samples was below 10.9%. The RSD of recovery was between 1.5% and 12.1%, which met the criteria.

**TABLE 5:** Matrix effects and recovery for the 15 ATBs.

Analytes	Conc. (μg/mL)		Recovery (n = 6)		IS-normalized matrix factor (n = 3 × 6)	
			Mean (%)	Precision (RSD %)	Mean (%)	Precision (RSD %)
CAS	LQC	1.50	81.6	8.9	98.1	5.5
	MQC	8.00	88.1	9.4	93.2	3.8
	HQC	40.00	86.3	8.1	101.9	5.1
IPM	LQC	3.00	38.1	7.7	88.0	10.9
	MQC	16.00	36.1	4.8	101.3	8.2
	HQC	80.00	33.6	12.1	107.2	6.6
LZD	LQC	0.75	106.4	6.2	97.9	4.6
	MQC	4.00	106.8	4.7	97.3	3.8
	HQC	20.00	103.1	4.0	111.8	3.2
MEM	LQC	3.00	92.1	12.0	104.7	3.6
	MQC	16.00	92.5	8.3	93.9	4.4
	HQC	80.00	94.7	3.8	100.2	5.1
MOX	LQC	3.00	32.2	10.1	106.9	1.5
	MQC	16.00	31.5	3.4	95.9	5.5
	HQC	80.00	33.5	5.1	104.7	8.6
PRL	LQC	3.00	97.8	4.9	93.1	2.9
	MQC	16.00	96.4	1.5	97.5	1.1
	HQC	80.00	96.9	5.2	103.3	2.1
VCZ	LQC	0.30	102.8	6.5	100.6	3.0
	MQC	1.60	103.5	4.3	95.5	3.8
	HQC	8.00	101.9	4.4	104.3	3.7
POS	LQC	1.50	75.8	7.5	95.3	5.4
	MQC	8.00	84.1	7.5	92.0	8.2
	HQC	40.00	79.2	8.4	96.6	3.1
CMZ	LQC	3.00	92.1	8.2	108.3	3.7
	MQC	16.00	100.8	6.4	99.1	5.0
	HQC	80.00	85.7	6.8	103.6	2.0
TIG	LQC	1.50	106.0	9.9	102.1	9.8
	MQC	8.00	97.8	7.3	99.1	6.8
	HQC	40.00	96.2	7.3	113.5	7.3
CFZ	LQC	3.00	92.8	10.2	106.9	6.9
	MQC	16.00	97.1	6.1	97.2	3.8
	HQC	80.00	93.9	4.4	104.4	5.1

#### 3.2.5 Stability

The stability results of all ATBs are summarized in Table 6. In plasma stored at room temperature, IPM resulted in the most unstable drug, with a loss percentage at 4 h > 15%. After 8 h, a degradation trend became evident for MEM, MOX, and PRL. The autosampler stability is limited for IPM, where significant degradation occurs after 24 h. All the other compounds remained stable in the autosampler at 4 °C up to 5 days. In a 4 °C refrigerator, the compounds in the plasma samples tested were stable for at least 24 h, except for IPM, which was only stable for 8 h. At −80 °C all drugs were stable for 37 days. Freeze and thaw stability results were within the acceptability ranges for all the drugs, except for IPM.

**TABLE 6:** Stability of 15 ATBs in human plasma.

Analytes	Nominal conc		Stability (%)			
	QC samples	(μg/mL)	Room temperature	Freeze-thaw	Long-term	Autosampler
			(25 °C, 18 h)	(−20 °C, Five cycles)	(−80 °C, 37 days)	(4 °C, 6 days)
CAS	LQC	1.50	96.8	97.3	104.7	102.0
	HQC	40.00	98.9	89.8	92.8	99.0
IPM	LQC	3.00	—	—	93.3	—
	HQC	80.00	22.3	—	94.3	—
LZD	LQC	0.75	102.9	105.6	105.1	102.9
	HQC	20.00	97.0	98.0	103.5	102.5
MEM	LQC	3.00	50.3	88.7	96.3	95.7
	HQC	80.00	65.7	91.6	89.4	95.7
MOX	LQC	3.00	86.2	89.0	96.0	99.0
	HQC	80.00	69.8	88.6	100.9	99.8
PRL	LQC	3.00	57.8	88.7	94.7	106.0
	HQC	80.00	56.3	89.9	92.4	106.1
VCZ	LQC	0.30	90.5	97.3	92.7	103.3
	HQC	8.00	97.2	97.4	101.5	105.0
POS	LQC	1.50	104.2	94.0	108.7	101.3
	HQC	40.00	104.8	95.3	114.5	97.0
CMZ	LQC	3.00	82.4	105.0	81.7	106.0
	HQC	80.00	76.8	99.0	90.8	99.4
TIG	LQC	1.50	108.3	100.7	113.3	98.7
	HQC	40.00	114.9	88.0	95.5	98.0
CFZ	LQC	3.00	88.6	95.0	102.7	105.7
	HQC	80.00	84.2	96.6	96.0	99.8

### 3.3 Application to clinical samples for routine TDM

The validated method was routinely applied to the concentration monitoring of ATBs in 26 plasma samples from 16 patients treated at the Children’s Hospital of Nanjing Medical University (Table 7). Some patients were under a combined therapy, and their samples contained two antibiotics simultaneously. Among the 25 samples, LZD was present in 24 of them; the concentration ranged from 0.03 to 11.7 μg/mL for LZD. The lowest value for LZD was actually below the LLOQ (0.25 μg/mL). Nevertheless, we consider that the clinical significance of these low levels was essentially the same. At the same time, the Internal Quality Control practice of MEM, IPM, LZD, and TIG has also been qualified, which was organized by the National Center for Clinical Laboratories (NCCL).

**TABLE 7:** Details of patients with infection and drug through concentration (*C*_min_) in plasma.

Patients	Age (Year)	Infection type	Analytes	Dosage (g/24 h)	ATBs concentration (μg/mL)
1	9	Soft tissue infection	LZD	1.20	8.25
2	15	Osteomyelitis	LZD	1.20	0.92/4.69
3	12	Pelvic abscess	LZD	1.20	11.70
4	1	Meningitis	LZD	0.27	0.03/0.88
5	11	Osteomyelitis	LZD	1.80	5.10
6	2	Sepsis	LZD	0.35	0.19
7	0.1	Pneumonia	LZD	0.09	1.75
8	0.3	Pneumonia	LZD	0.26	2.58
9	7	Coxitis	LZD	0.90	1.40/4.66
10	8	Osteomyelitis	LZD	1.11	3.34
11	0.1	Sepsis	LZD	0.13	0.81
12	5	Pneumonia	LZD	0.60	2.95/3.83
13	1	Arthrophlogosis	LZD	0.30	0.74/0.19/1.99/2.98/7.87/0.057
14	12	HSCT	POS	0.30	1.75/1.50
15	0.1	Pneumonia	MEM	0.36	66.20
16	0.1	Sepsis	MOX	0.24	34.40

Abbreviation: HSCT, hematopoietic stem cell transplantation.

## 4 Discussion

Indeed, blood-based TDM has the potential to optimize antimicrobial concentrations through personalized dosing, maximizing therapeutic efficacy, minimizing drug-related toxicity, and mitigating the development of antimicrobial resistance (Brasier et al., 2023). Of course, the optimal analytical method is a prerequisite for achieving these clinical objectives. Impressively, in the current study, we have developed and validated a LC-ESI-MS/MS method for the simultaneous determination of 11 antibiotics. Although several reports exist (Lu et al., 2022; Barco et al., 2020), our method still holds advantages in terms of sample consumption, sample pretreatment, analysis time, and linear range. Of note, our method’s development involved resolving critical analytical hurdles, thereby facilitating accurate multi-antimicrobial quantification.

Firstly, for column selection, we decided to use reverse-phase chromatography based on C18 chemistry in accordance with many other methods (Table 1). Given the relatively high polarity of β-lactam antibiotics, we initially attempted to separate these agents using a HILIC column (4.6 mm × 50 mm, 3 μm) and Waters (4.6 mm × 75 mm, 3.5 μm) (Kahsay et al., 2014; Rehm and Rentsch, 2020). However, compounds such as CAS and TIG, which exhibit extremely high hydrophobicity (logP >3), showed negligible retention under HILIC conditions. This phenomenon was likely attributed to the strong hydrophobic interactions or hydrogen bonding between the hydrophobic side chains of CAS (*e.g.*, the myristoyl group) and residual silanol or amino groups on the HILIC stationary phase (Jandera, 2011), leading to irreversible adsorption and failure to elute detectable peaks.

To address this challenge, we tested various chromatographic columns packed with core-shell stationary phases for the analysis. The core-shell design minimizes longitudinal diffusion (*B* term in the Van Deemter equation) to reduce peak broadening, while its reduced eddy diffusion (*A* term) and enhanced mass transfer kinetics (*C* term) collectively yield flatter Van Deemter curves to enhance column efficiency (theoretical plates increased by ∼30%). This is quantitatively described by: *H = A + B/u + C·u*, allowing for operation at higher flow rates (0.5 mL/min) without significant loss of efficiency (Hayes et al., 2014; Gritti et al., 2010). By optimizing a gradient elution program starting with a high aqueous phase (*e.g.*, 92% H_2_O containing 0.1% FA) and gradually increasing the organic phase (ACN), we achieved stable separation of antibiotics with wide polarity ranges. For instance, polar β-lactams (*e.g.*, IPM, logP ∼1.82) were retained early in the run, while highly hydrophobic compounds like CAS eluted later with sharp peaks (asymmetry factor <1.2), demonstrating robust resolution and reproducibility.

In addition, chromatographic performance depends critically on column geometry and particle characteristics. While longer columns (*e.g.*, 100 or 150 mm) enhance resolution through increased phase interactions, they proportionally extend analysis time. Similarly, smaller particles (*e.g.*, <3 μm) improved efficiency but required higher operating pressures (Nguyen et al., 2006). Following parameter evaluation, a Kinetex C18 column (2.1 × 75 mm; 2.6 μm) was selected to optimally balance chromatographic separation efficiency with practical analytical constraints.

Secondly, carryover is a prevalent issue in LC-ESI-MS/MS analysis, which can significantly impact the accuracy and reliability of analytical results. Carryover can originate from the autosampler, switching system, and the liquid chromatography column (Hughes et al., 2007). Indeed, we encountered significant carryover challenges during the development of this assay. Impressively, the carryover contamination from the autosampler and separation column was systematically identified and eliminated. A more thorough auto-sampler flushing (before and after rising) and washing procedures using different solvents (MeOH, ACN, 50%MeOH, and ACN:MeOH:IPA:H_2_O (4:4:1:1, v/v/v/v)) were tested. Following unsuccessful autosampler washing procedures, column-derived carryover was investigated via duplicate solvent gradient analysis.

Interestingly, we observed significant column memory effects for MOX. To address this issue, we modified the elution gradient by increasing the initial proportion of the organic phase and extending the elution time. Despite these efforts, chromatographic memory effects persisted, yielding MOX carryover peaks at approximately 70% of corresponding analyte signals. Such high carryover rates posed a significant challenge, especially when analyzing low-concentration samples following high-concentration ones, as it could introduce substantial deviations in the analytical results.

One approach to minimize carry-over involves sequential blank injections interspersed between sample analyses. While sequential blank injections progressively reduce carry-over peaks, they concomitantly increase analytical run times, rendering this approach suboptimal for high-throughput workflows. To achieve further carryover mitigation, a CE defect strategy was implemented (Shen et al., 2021). During method optimization, MOX intensity exceeding 10^5^ cps prompted incremental CE reduction (5 eV steps from 20 to 10 eV). At 50% CE reduction (10 eV), the maximum MOX response decreased to 10^4^ cps, with residual carryover reduced to 18% – satisfying predefined acceptance criteria.

Finally, given the well-documented instability of β-lactam antibiotics, particularly in biological matrix, stringent sample handling protocols were implemented throughout the investigation. This included sample transportation, pre-analytical, and analytical process. Consistent with previous reports (Dailly et al., 2011), our stability studies revealed that significant degradation of IPM at ambient temperature, with observable analyte loss occurring within 2 h. Other β-lactams (like MOX, MEM, and PRL) showed a degradation trend after 4 and 6 h, respectively (Figure 4). To ensure analyte integrity, the following measures were strictly enforced: (1) all specimens were maintained at 0 °C–4 °C during transportation using ice-packed containers; (2) time from collection to laboratory processing was constrained to <2 h; (3) immediate centrifugation was conducted at 4 °C (3,000 × *g*, 10 min) upon arrival; and (4) the separated plasma was then immediately frozen at −80 °C for further analysis. Indeed, these steps maintained >95% analyte stability throughout the sample handling workflow. Thus, the 2-h processing window was selected based on degradation kinetics while accommodating typical clinical logistics.

Compared to existing assays, our study demonstrates obvious advantages in three key aspects: (1) By overcoming the inherent limitation of HILIC in retaining hydrophobic analytes while fully leveraging the high efficiency and versatility of core-shell columns, we developed a novel one-step precipitation method enabling simultaneous detection of 11 structurally diverse antimicrobial agents. (2) We propose comprehensive implementation of stringent sample-handling protocols throughout the analytical workflow to minimize pre-analytical degradation during transport and processing, thereby enhancing measurement accuracy in real-world clinical settings. (3) Crucially, our established synchronous quantification method for these 11 antimicrobials meets all critical internal validation criteria—including precision, accuracy, linearity, and stability. Furthermore, external validation conducted through the NCCL proficiency testing program confirmed the method’s reliability for key antibiotics, including MEM, IPM, LZD, and TIG.

Certainly, our study also has its limitations. We have refined the extraction process of the samples and conducted a comparison of the extraction effects using different precipitants (MeOH, ACN, MeOH: ACN, ACN containing 0.1% FA, and MeOH containing 0.1% FA). Our results did not show significant differences in extraction recovery rates. In our study, the recovery of IPM was only about 40% (Table 4). As reported by Ohmori *et al.*, the recovery for doripenem was found to be approximately 50%, whereas the extraction rates for other compounds were consistently above 80% (Colin et al., 2013). Given these findings, it was evident that alternative sample preparation techniques should be explored to enhance the recovery efficiency of individual compounds in the samples. Despite the observed low recovery IPM in this work, these limitations did not affect its quantitative analysis, owing to the high specificity and sensitivity of LC-ESI-MS/MS. The simple sample pretreatment process further improves its compatibility with clinical sample analysis.

In the clinical application, due to the nature of the available clinical samples, we only tested the plasma concentrations of LZD, POS, MEM, and MOX. Therefore, it was impossible to comprehensively evaluate the clinical performance of the newly established method, which might affect the general applicability of the validation results across all analytes. To address this issue, we will implement this method in routine clinical practice and optimize the assay with larger size samples.

## 5 Conclusion

A rapid and sensitive LC-ESI-MS/MS method for the simultaneous determination of 11 ATBs (CAS, IPM, LZD, MEM, MOX, PRL, VCZ, POS, CMZ, TIG, and CFZ) has been presented. The validated method has been proven to be accurate and precise with a simple protein precipitation procedure. Moreover, the reproducible method was successfully used for monitoring 6 ATBs (LZD, MEM, MOX, POS, IPM, TIG) in clinical practice. The concentration of ATBs could be quantified using small volumes of human plasma (10 μL), and it is significantly important for pediatric patients undergoing polytherapy. Therefore, the determination of 11 ATBs levels can provide valuable information for pediatricians to implement individualized treatment for children and reduce the occurrence of antibiotic resistance.

## Data Availability

The original contributions presented in the study are included in the article/supplementary material, further inquiries can be directed to the corresponding authors.
